# Expression of the mRNA stability regulator Tristetraprolin is required for lactation maintenance in the mouse mammary gland

**DOI:** 10.18632/oncotarget.23904

**Published:** 2018-01-03

**Authors:** María Victoria Goddio, Albana Gattelli, Johanna M. Tocci, Lourdes Pérez Cuervo, Micaela Stedile, Deborah J. Stumpo, Nancy E. Hynes, Perry J. Blackshear, Roberto P. Meiss, Edith C. Kordon

**Affiliations:** ^1^ IFIBYNE-UBA-CONICET, Departamento de Química Biológica, FCEN-UBA, Buenos Aires, Argentina; ^2^ Friedrich Miescher Institute for Biomedical Research, Basel, Switzerland; ^3^ Signal Transduction Laboratory, National Institute of Environmental Health Sciences, Research Triangle Park, Durham, North Carolina, USA; ^4^ National Academy of Medicine, Buenos Aires, Argentina

**Keywords:** lactation, Tristetraprolin, TNF alpha, cell death, mammary involution, Autophagy

## Abstract

Tristetraprolin (TTP), an mRNA-binding protein that negatively controls levels of inflammatory factors, is highly expressed in the lactating mouse mammary gland. To determine the biological relevance of this expression profile, we developed bi-transgenic mice in which this protein is specifically down-regulated in the secretory mammary epithelium in the secretory mammary epithelium during lactation. Our data show that TTP conditional KO mice produced underweight litters, possibly due to massive mammary cell death induced during lactation without the requirement of additional stimuli. This effect was linked to overexpression of inflammatory cytokines, activation of STAT3 and down-regulation of AKT phosphorylation. Importantly, blocking TNFα activity in the lactating conditional TTP KO mice inhibited cell death and similar effects were observed when this treatment was applied to wild-type animals during 48 h after weaning. Therefore, our results demonstrate that during lactation TTP wards off early involution by preventing the increase of local inflammatory factors. In addition, our data reveal the relevance of locally secreted TNFα for triggering programmed cell death after weaning.

## INTRODUCTION

The stability of many mRNAs is controlled by AU-rich elements (AREs), located within the 3’UTR of their transcripts [1, 2]. ARE-directed control of mRNA decay is mediated through ARE-binding proteins. One such protein is Tristetraprolin (TTP), coded by *Zfp36* gene, which accelerates decay of targeted transcripts [3, 4]. It has been shown that TTP-KO mice exhibit severe chronic inflammation in multiple tissues, mostly due to a dramatic increase of TNFα levels [5]. Besides, it has been demonstrated that TTP reduces expression of many other cytokines and oncoproteins [6] and can also participate in glucocorticoid-mediated anti-inflammatory activity [7, 8]. Specifically in mammary epithelial cells, we demonstrated that TTP expression is regulated by lactogenic hormones, reaching its highest expression in the gland when mammary epithelium differentiation is final [9].

At lactation, final differentiation of mammary epithelium occurs concomitantly with activation of STAT-5, a Prolactin-activated transcription factor that is essential for lactogenic hormone response [10]. This factor synergizes with the glucocorticoid receptor (GR) to induce milk protein expression and cell survival [11]. After weaning, a dramatic switch towards death signaling leads to mammary gland involution. This process is also characterized by extensive tissue remodeling to return the gland to a pre-pregnant state [12, 13]. In mice, natural involution starts approximately three weeks after delivery, and cell death is largely completed by day 24 [14]. To reproducibly test mechanisms involved in mouse mammary involution, lactation is abruptly interrupted during the first 10 days after delivery, following procedures such as teat sealing [15], unilateral cessation of milking of a single gland [16] or abrupt removal of pups [17]. These approaches have revealed that expression of inflammatory cytokines, such as IL6 [18], LIF [19, 20] and TNFα [21], is dramatically increased upon weaning. In turn, these cytokines regulate transcription through factors as STAT3 and NFκB, which play pivotal roles in determining mammary cell fate [22–25].

Until a few years ago, mammary involution was assumed to be driven solely by apoptosis of secretory epithelium. More recently, it has been shown that early cell death (24 h post forced weaning) occurs through an initial phase of lysosomal-mediated cell death [26, 27] followed by apoptosis [28, 29]. Upon weaning, lysosomal membrane permeabilization occurs, resulting in up-regulation and leakage of lysosomal contents, such as cathepsins, which act as “executioner proteases” [26].

Here, we assess TTP role during lactation by analyzing the phenotype of a conditional KO mouse in which TTP expression is dramatically reduced in mammary glands of lactating animals. Our results show the relevance of TTP in lactation maintenance and the important role of locally produced TNFα in triggering programmed cell death in the mammary epithelium after weaning. In addition, as it has been proposed that post-partum breast involution may be responsible for the increased metastatic potential of post-partum breast cancer [30], we hypothesize that the assigned tumor suppressor role of TTP in the mammary gland [9, 31] might be related to its ability to prevent involution associated events in this tissue.

## RESULTS AND DISCUSSION

**Figure 1:**
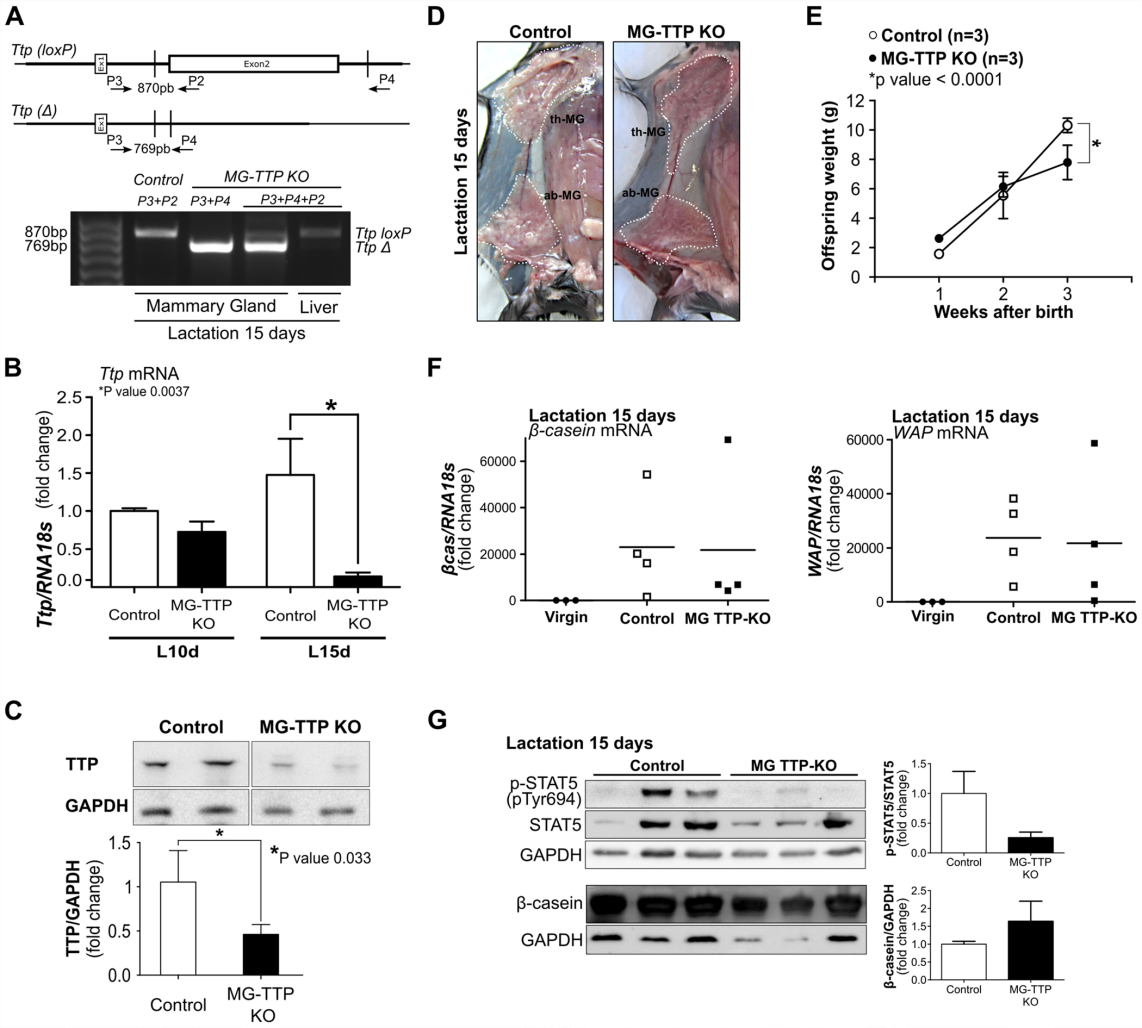
Mammary-specific TTP down-regulation leads to lactation deficiency (**A**) Upper panel shows maps of loxed-*Ttp* gene (*Ttp*(*loxP*)) and its recombined version (*Ttp*(Δ)) after Cre-mediated recombination (adapted from Qiu et al. 2012). Open box represents 2^nd^ coding exon. Primer (P2, P3, P4) locations, used for genotyping, are indicated by arrows. Lower panel shows PCR analysis of genomic DNA to detect *Ttp*-loxP gene and *Ttp* Cre-mediated deletion (TtpΔ) in mammary gland and liver from female Control and MG-TTP KO mice. (**B**) RT-qPCR analysis of *Ttp* mRNA in mammary glands from MG-TTP KO and Control females at 10 days (L10d) and 15 days (L15d) of lactation (*n* = 4). (**C**) Representative images and quantification of WB analysis of TTP protein expression in mammary glands of MG-TTP KO and Control mice at L15d; each column shows mean+s.e.m. (*n* = 4). (**D**) Examples right #4 mammary glands of MG-TTP KO and Control mice at L15d; dotted line delimits #2 and #3 thoracic glands (th-MG), and #4 abdominal gland (ab-MG). (**E**) Offspring weight from MG-TTP KO (●) or Control (○) mothers; each point represents mean+s.e.m. of 3 litters (6-8 pups each). (**F**) RT-qPCR analysis of β-casein and *WAP* mRNA expression in mammary glands from virgin Control (Virgin), and Control and MG-TTP KO mice at L15d; each point shows fold change compared to Virgin mice and represents a different mouse. (**G**) Representative images and quantification of WB analysis from Control and MG-TTP KO mammary glands at L15d with the indicated antibodies; each column shows mean + s.e.m (*n* = 5).

In epithelial cells of mammary gland TTP conditional KO mice (MG-TTP KO), Cre-mediated recombination of loxed *Ttp* was clearly observed at 15 days of lactation (Figure 1A) and, accordingly, *Ttp* mRNA (Figure 1B) and protein (Figure 1C) levels were reduced in those glands by approximately one third and half, respectively. Even before significant changes in TTP levels were detected, morphological differences between experimental and control mammary glands were observed. Histological analysis of MG-TTP KO glands at 10 day of lactation showed that the acini were formed by a layer of epithelial cells with hyperchromatic nuclei, often displaced to the cell center, while in control animals nuclei were mainly in the basal region (Supplementary Figure 1A a&b). In addition, acini from bi-transgenic mouse glands were lined by a thinner basement membrane, compared to controls (Supplementary Figure 1A c&d). However, at that time no differences were detected in STAT5A activation or β-casein protein levels between experimental and control animals (Supplementary Figure 1B).

At 15 days of lactation, MG-TTP KO glands showed less milk accumulation than glands from control mice (Figure 1D). This observation suggested that a decreased milk production might be the reason for the reduced average weight detected in the MG-TTP KO offspring at the end of lactation (Figure 1E). Although no significant differences between experimental and control mice were found in β*-casein* and *WAP* mRNA levels and in β*-casein* protein expression, STAT5 phosphorylation (P-STAT5) was homogeneously low in experimental glands at this time (Figure 1F and 1G). We understand that variability of milk protein mRNA levels observed among each group was due to natural declining of milk production, which Eliminate naturally occurs in the third week of lactation (also shown by the variable levels of P-STAT5 in the control group). In fact, it has been established that a sharp drop in milk yield naturally happens at 15 days of lactation, concomitantly with pups initiating dry food consumption [32].

**Figure 2:**
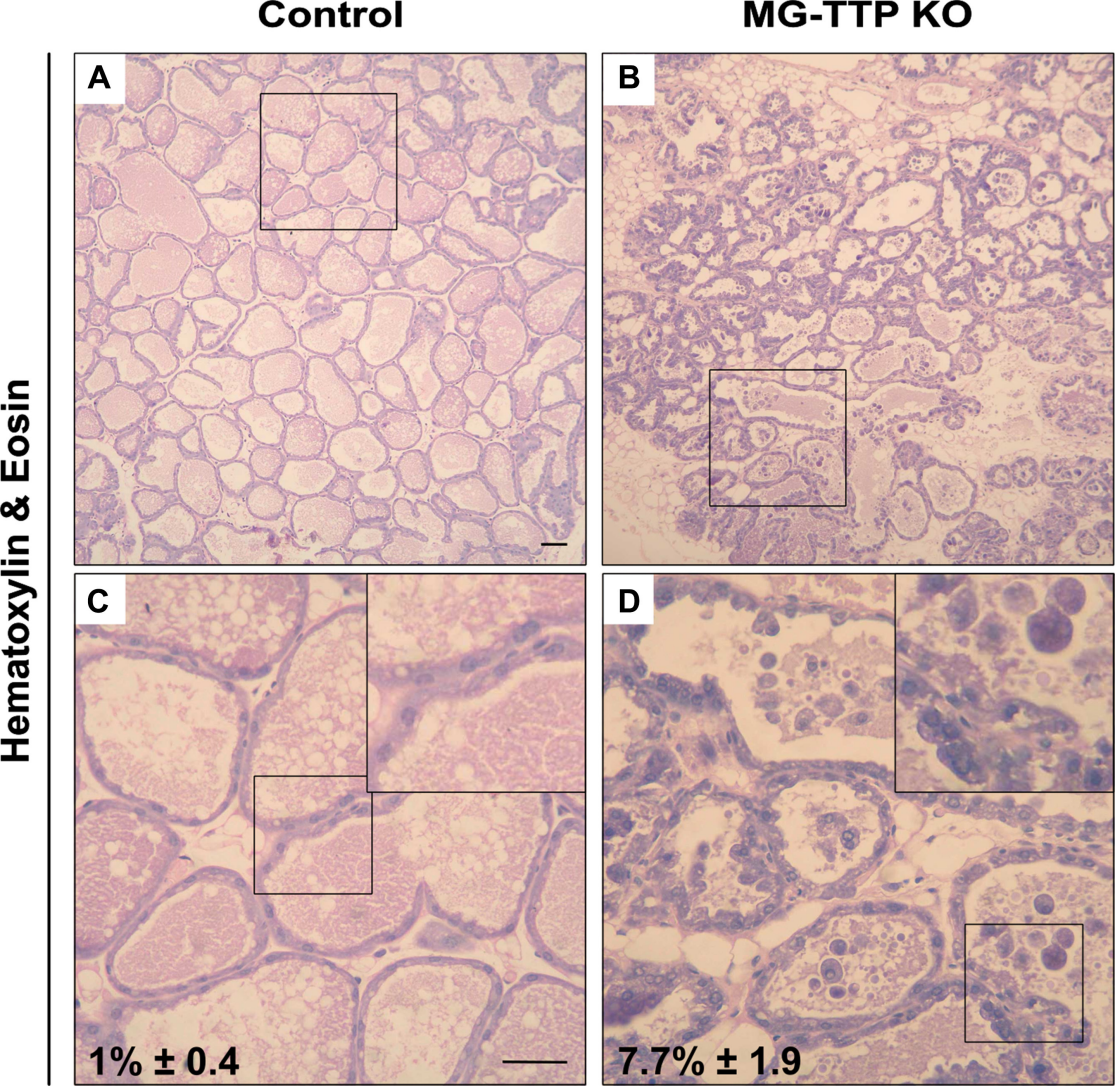
Loss of TTP expression in lactating mammary glands induces cell death Representative images of H&E staining in mammary gland from control and MG-TTP KO: (**A** and **B)** original magnification 100X. (**C** and **D**), original magnification: 400X; c&d magnification of regions framed in a&b; insets: framed regions in c&d, respectively; percentages of apoptotic-like cells in H&E stained samples are indicated, mean + s.e.m; *n* = 3; *p* = 0.013 in c&d.

**Figure 3:**
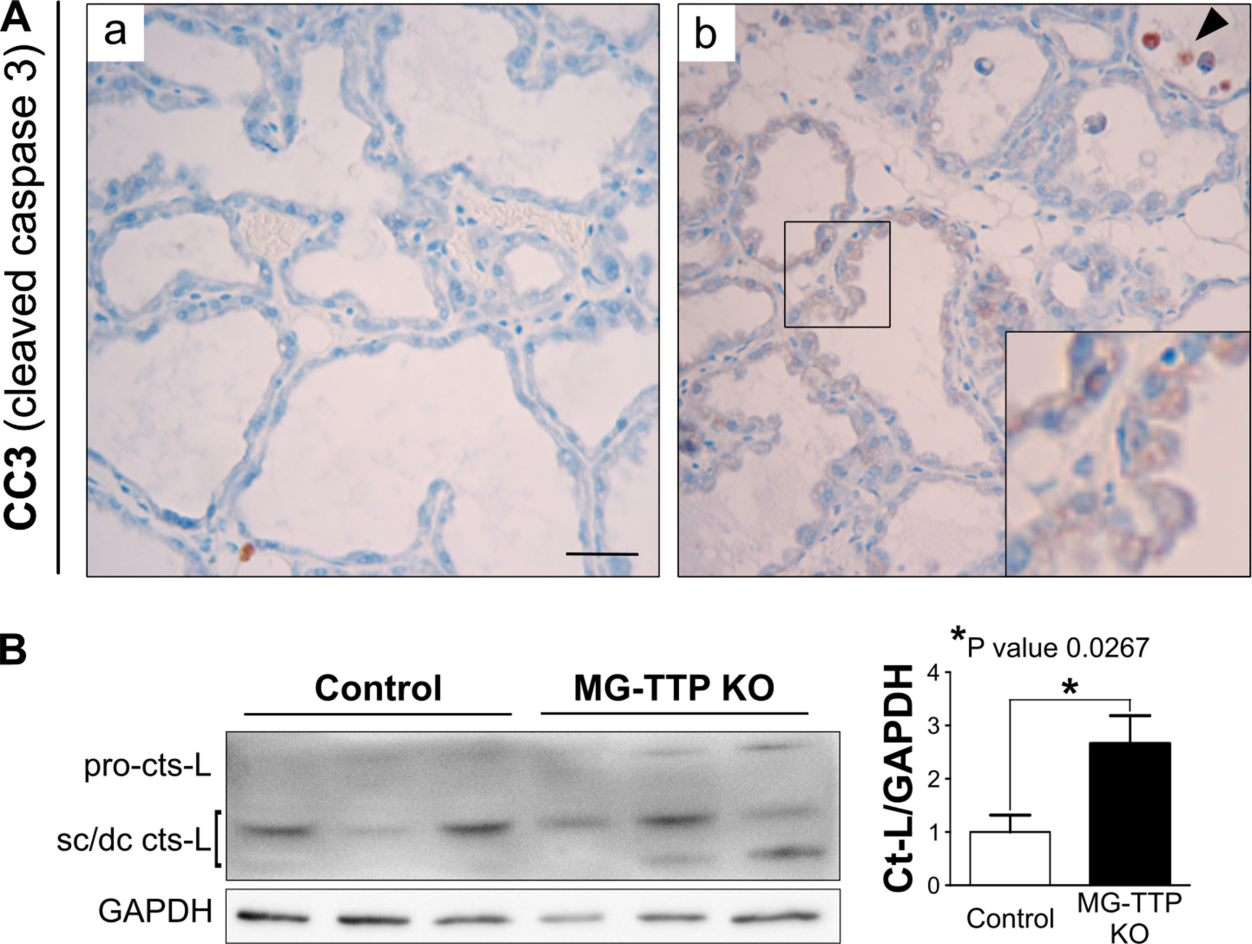
Cleaved caspase 3 (CC3) and active Cathepsin-L in MG-TTP KO lactating glands (**A**) Representative images of CC3-IHC in mammary glands from Control and MG-TTP KO at 15 days of lactation; original magnification: 400X. In b) CC3 positive stained cells attached to the extracellular matrix are shown amplified in the inset. Arrowhead indicates CC3 positive cells into alveolar lumens. Scale bar 100 µm. (**B**) Representative image and quantification (mean+s.e.m., *n* = 3) of WB analysis showing the increase of Cathepsin-L (cts-L; sc, single chain, dc, heavy chain of the double-chain form) from Control and MG-TTP KO mammary glands at 15 days of lactation (*n* = 3).

At histological level, the 15-day lactating mammary glands of MG-TTP KO showed clearly altered morphology, compared to controls. They presented irregularly shaped acini containing a pseudostratified epithelium with columnar cells showing supranuclear vacuoles protruding into the lumen (Figure 2D and Supplementary Figure 2C and 2D). MG-TTP KO glands also exhibited foci of intra-lobular adipose tissue that, although scanty, suggested initiation of involution-associated remodeling of the gland. In addition, a significant increase of apoptotic-like cells (rounded cells with hyperchromatic nuclei and intensely eosinophilic cytoplasm) was detected in the lumens (Figure 2D and Supplementary Figure 2D). Consequently, active (cleaved) caspase-3 (CC3)-labeled cells (both shed and attached to the acini) (Figure 3A) were observed at that time in MG-TTP KO glands. Noteworthy, it has been reported that alveoli of wild type (WT) mice remain intact up to day 18 of lactation, and evidence of nuclear karyorrhexis is only observed at day 22, during natural weaning [14]. In addition, CC3 labeling is not detected in shed cells before 12 h and in the alveolar wall only 72 h after forced weaning [29]. Besides, MG-TTP KO lactating glands showed an increase of active cathepsin-L (Figure 3B) that has been previously associated with lysosomal cell death, a process that normally occurs shortly after forced weaning [26]. Although our data indicate that the single-chain increases more markedly than the double-chain isoform, for quantification we considered them together, since they are both active forms of this protein [26].

**Figure 4:**
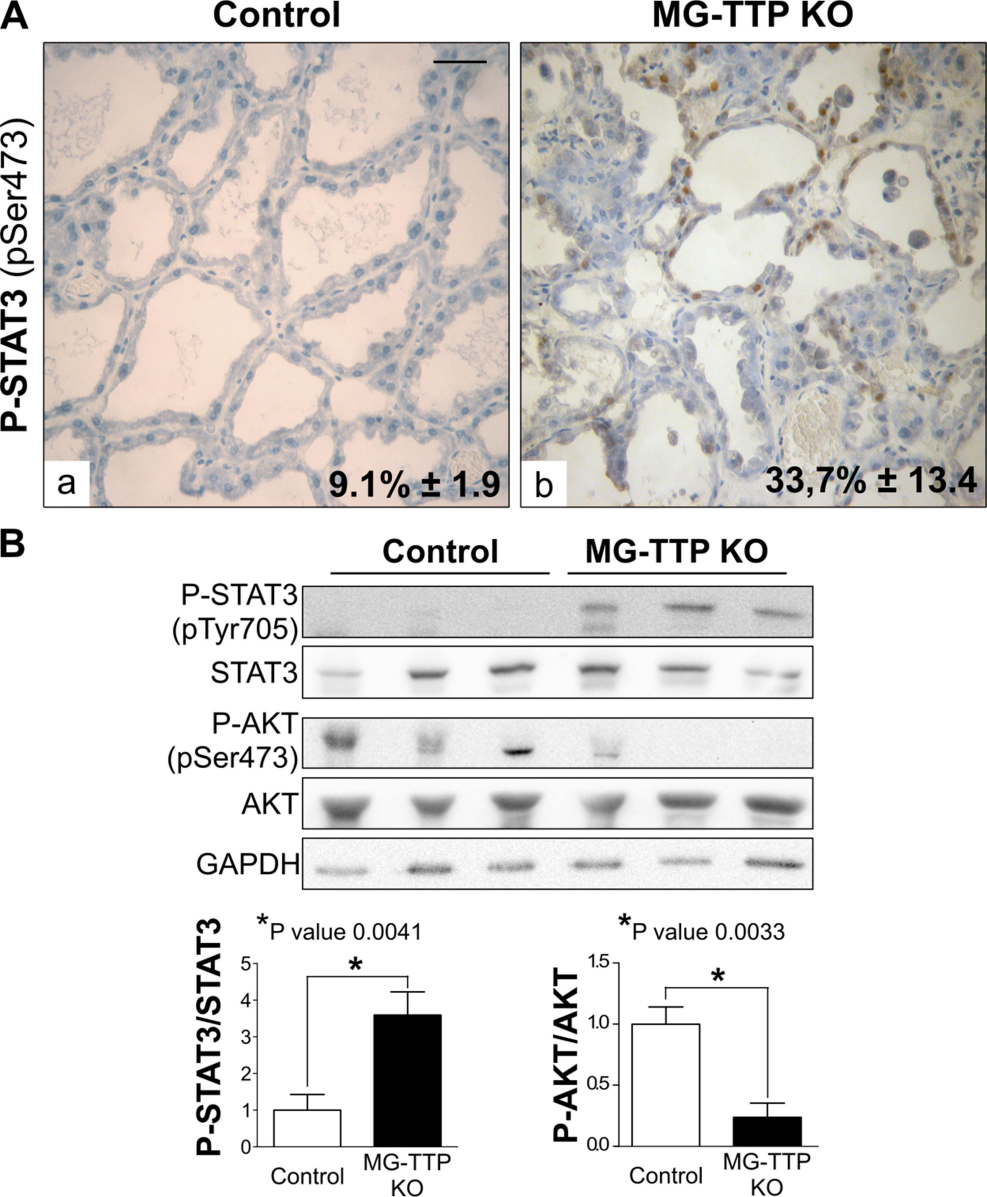
Life/Death signaling switch in 15-day lactating MG-TTP KO mice (**A**) Representative images of P-STAT3 IHC staining in mammary glands from Control and MG-TTP KO mice at 15 days of lactation. Original magnification: 400X. In a&b percentages of stained nuclei are shown in the lower right corner: mean+s.e.m. *n* = 3; *p* < 0.0001. Scale bar 100 µm. (**B**) Representative images (*n* = 3) and quantification (mean+s.e.m., *n* = 5) of WB analysis for P-STAT3, STAT3, P-AKT, AKT and GAPDH in Control and MG-TTP KO mammary glands at 15 days of lactation.

Upon forced weaning, mammary cell death is associated with down regulation of P-AKT signaling reportedly caused by STAT3 activation, which induces PI3K regulatory subunits p50α/p55α [33, 34]. Our results show that similar pathways were induced by TTP down-regulation, since mammary glands from bi-transgenic females showed higher P-STAT3 and lower P-AKT compared to controls at day 15 of lactation (Figure 4A and 4B). Moreover, it has been shown that prolonged cell survival through sustained up-regulation of *Akt1* occurs in response to STAT5 gain-of-function despite STAT3 activation in animals with forced weaning [35]. In our model, control mammary glands showed quite variable P-STAT5 levels on day 15 of lactation (Figure 1G), while P-STAT3 and P-AKT levels were homogenous among mice of the same group, and increase of the former and decrease of the later were associated with cell death in experimental mice. Therefore, although inhibition of STAT5 activation might have contributed to early mammary cell death in MG-TTP KO mice, we hypothesize that down regulation of TTP leads more directly to STAT3 activation, which would be responsible for reduction of P-AKT levels and early induction of mammary involution associated events.

**Figure 5:**
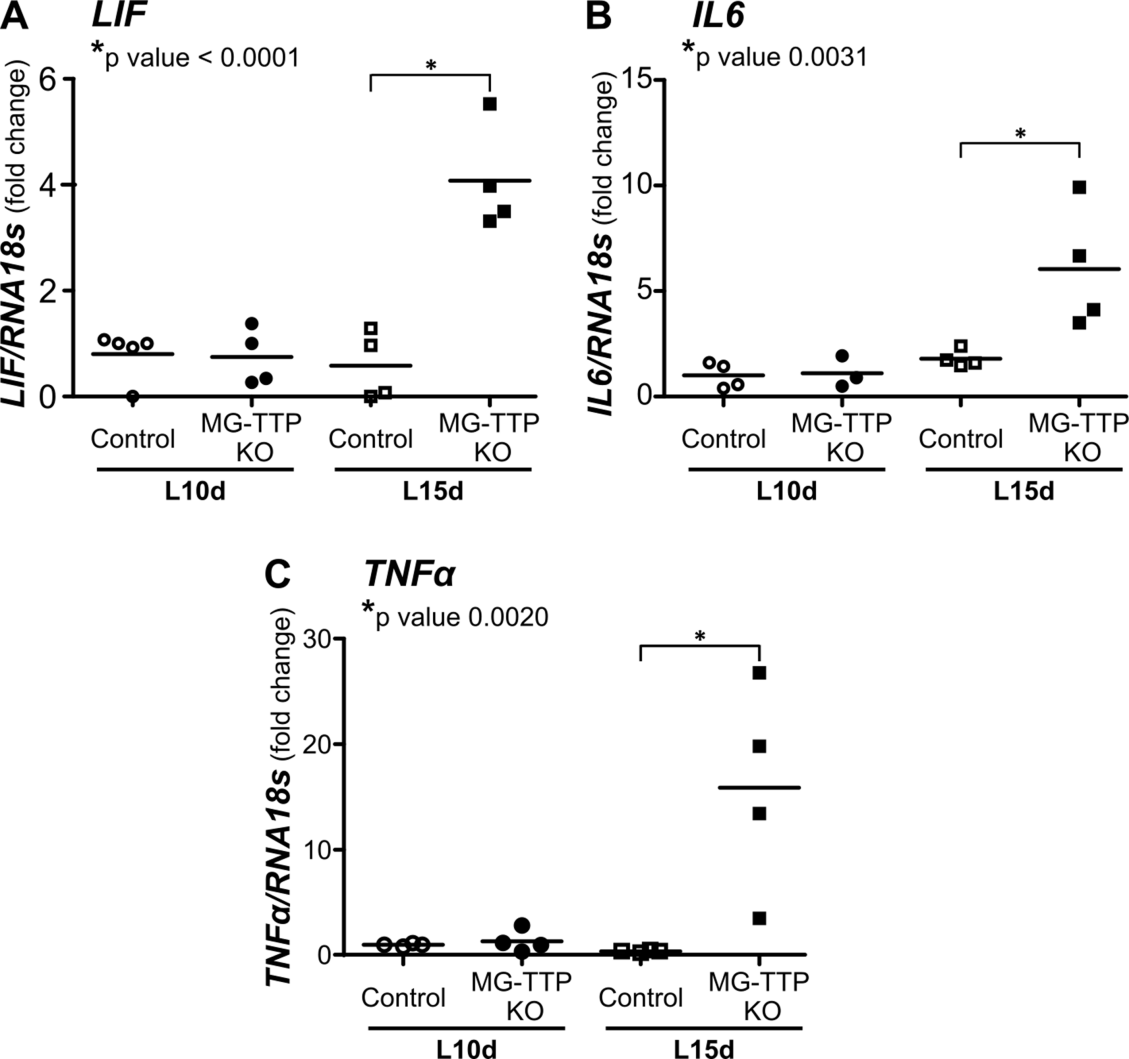
Increase of inflammatory cytokine mRNA levels in 15-day lactating MG-TTP KO mice RT-qPCR analysis of *LIF*, *IL6* and *TNFα* levels normalized to *18S* RNA; fold changes compared to control at L10d are shown. L10d: 10 day lactating; L15d: 15 day lactating mice. Each point corresponds to a different mouse and the stripe corresponds to the average value in each group. *P* values of statistical differences between Control and MG-TTP KO at L15d are shown for each cytokine.

Since it has been demonstrated that STAT3 activation in mammary gland is mainly triggered by LIF [19, 20], we wondered whether this cytokine might be involved in MG-TTP KO phenotype. Figure 5A shows that *LIF* expression was highly induced in lactating glands of experimental mice, as well as *IL6* and *TNFα* (Figure 5B and 5C). Importantly, these three cytokines are known targets of TTP destabilizing activity [4, 36, 37] and we have previously demonstrated that TNFα induces LIF expression in mammary cells [38]. Therefore, at the second week of lactation, we found that TTP down–regulation leads to an increase of inflammatory mediators associated with LIF/STAT3 signaling and components of death receptor pathways of apoptosis, which are present in mouse mammary glands at the very first phase of forced involution [21], but are normally absent at the initiation of natural weaning [39].

Our data clearly show that cellular events associated with forced-involution appear in lactating MG-TTP KO glands without any stimulus other than those triggered by TTP down-regulation. We propose that early initiation of mammary regression in the bi-transgenic females may account for milk production deficits and consequent underdevelopment of their pups. Interestingly, preliminary observations of mammary glands from 15-day lactating MG-TTP KO females in their 3rd lactation displayed similar histological features to those described above. However, after multiple pregnancies the bi-transgenic glands showed more intra-lobular adipose tissue and smaller acini, with less apoptotic-like cells in their lumens than glands from these mice during their first lactation. These observations may indicate that after successive pregnancies, MG-TTP KO glands start involution even earlier, so these features show a more profound and advanced involution-like process. Another possibility is that parity-induced mammary epithelial cells (PI-MEC), which act as lobule-limited epithelial stem/progenitor cells [40], might be affected by TTP loss. Then, alveolar development would become more defective in subsequent pregnancies. New experiments are being carried out to analyze these two possibilities.

To our knowledge, there is no evidence that TTP directly represses transcription of inflammatory cytokines, but a molecular model for understanding regulation of TNFα biosynthesis through two Post-transcriptional loops has been proposed. One loop is based on the positive feed-back generated by TNFα itself mediating expression of its own transcript, while the negative feedback would be due to TTP-mediated degradation of *TNF*α mRNA and TNFα-mediated expression of *Ttp* transcript [4, 41]. In addition, TTP might be repressing the initiation of inflammatory pathways acting together with glucocorticoids. These hormones, which are necessary for inducing milk protein expression in mammary gland [42], require TTP for repressing cytokine transcription in different cell types [7]. Therefore, we propose that in our experimental model, reduced expression of TTP would result in inhibition of glucocorticoid anti-inflammatory surveillance as well as in the increased half-life of inflammatory cytokine mRNAs that would be transcribed, albeit at low levels, during lactation.

**Figure 6:**
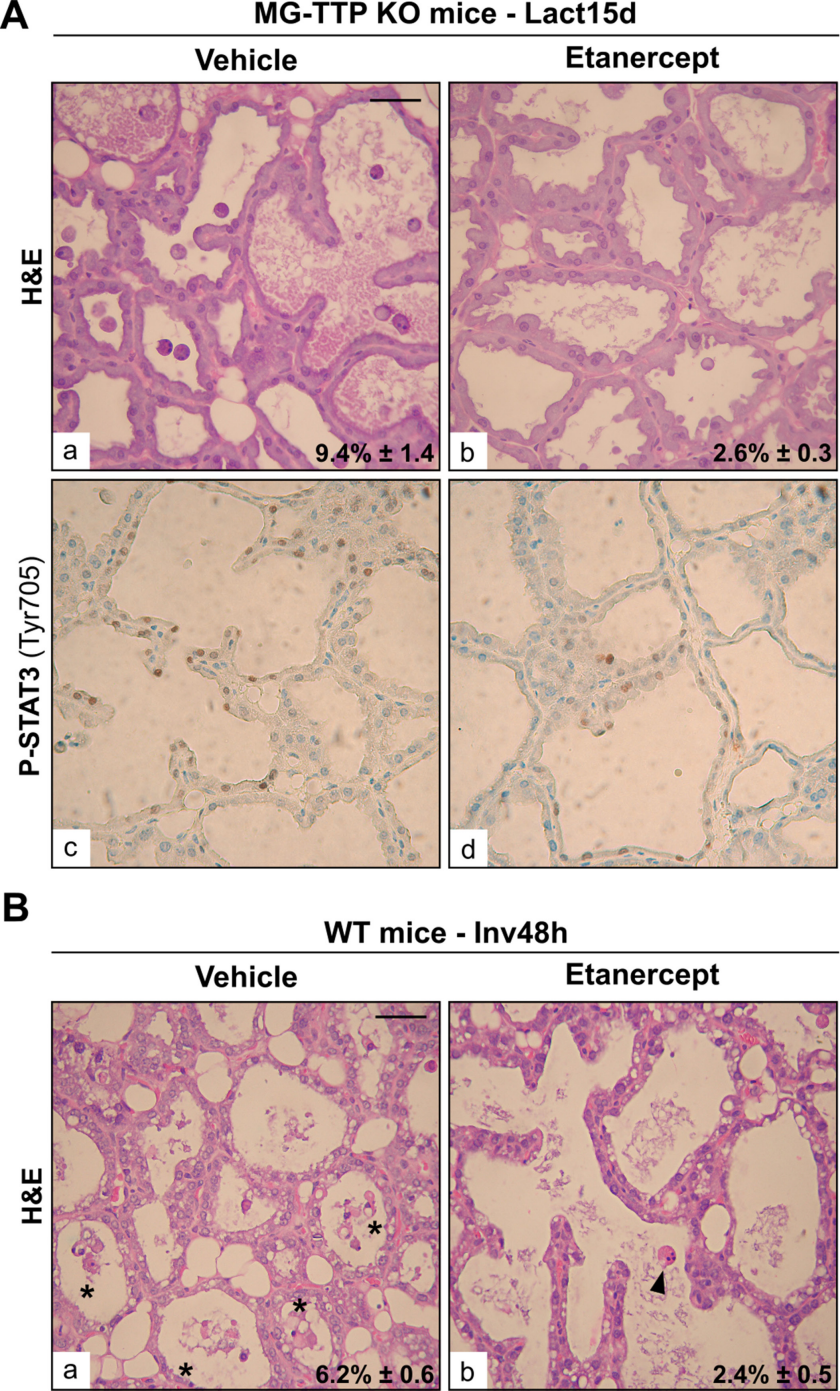
Etanercept treatment reduces mammary cell death (**A**) Representative images of H&E (a&b) and P-STAT3 IHC (c&d) of mammary gland sections from L15d MG-TTP KO mice treated with Etanercept or Vehicle. (**B**) Representative images of H&E (a&b) staining of mammary gland sections at 48 h of forced involution, from WT mice treated with Etanercept or Vehicle. In a) asterisks show clumps of apoptotic-like cells; b) arrowhead points to an individual apoptotic-like cell; in. In (A) and (B) percentages of dead cells detected in H&E panels are indicated (mean+s.e.m., *n* = 3); original magnification: 400X. Scale bar: 100 µm. In (A) *p* = 0.0084 and in (B) *p* = 0.0105.

It has been shown that treatment of young TTP-KO mice with antibodies that blocked TNFα prevented progress of essentially all aspects of their phenotype, which was mainly the development of inflammation and autoimmunity [5]. Similar results were observed in the offspring of TTP-KO mice bred with mice deficient in both types of *TNF*α receptors [43]. Therefore, we tested whether interfering with TNFα activity would be sufficient to rescue MG-TTP KO phenotype. Figure 6A shows that Etanercept, a recombinant fusion protein that acts as a TNFα decoy receptor, caused a significant reduction in the quantity of apoptotic-like cells found in alveolar lumens of bi-transgenic glands at 15 days of lactation compared to vehicle-treated MG-TTP KO mice. Besides, some regions of Etanercept-treated MG-TTP KO glands showed less P-STAT3 staining (Figure 6A c&d), and some of these animals displayed lower LIF and TNFα levels than those inoculated with vehicle (Supplementary Figure 3). Importantly, we have found that Etanercept treatment not only prevented massive cell death in MG-TTP-KO animals before weaning, but also exerted a similar effect in mammary epithelium of WT female mice 48 h after forced weaning (Figure 6B). This indicates that TNFα activity plays a central role in inducing mammary epithelial cell death in both MG-TTP KO and WT mice.

It is well known that TNFα promotes extrinsic apoptosis through caspase 8 activation [44]. However, we did not find significant differences in levels of cleaved-caspase 8 (Supplementary Figure 4A), IκBα (Supplementary Figure 4B) or p65 phosphorylation (data not shown), which are markers of NFκB activity, in MG-TTP KO compared to control lactating glands. However, Cathepsin-L active form increase (Figure 3B) may suggest induction of lisosomal cell death pathway. In agreement, a new mechanism has been proposed for TNFα as a mediator of cell death during mammary involution. It has been shown that this cytokine causes zinc (Zn) transporter ZnT2 redistribution, which leads to increased Zn in lysosomes, swelling of these organelles, cathepsin release, and lysosomal cell death [45, 46].

In summary, the major finding of this study is a completely new physiological role for the mRNA binding protein TTP, which has been described as a very important negative regulator of acute inflammation and pro-oncogenic events. Our results show that TTP is required for maintaining lactation, since its specific deficiency in mammary secretory cells results in the onset of early involution. The MG-TTP KO lactating glands displayed several features of wild type mammary epithelium after 24-48 h of forced weaning, such as induction of TNFα [21, 47], IL-6 family cytokines (IL-6 and LIF) [18–20, 48], STAT3 and caspase 3 activation, cell death induction [24, 25], and the decrease of P-AKT [33, 34]. Interestingly, in our model, induction of inflammatory signaling cascades did not require any mechanical or hormonal stimuli associated with weaning, or activation of the innate immune system. Besides, our approach allows us to distinguish, from the multiple pathways involved in cell-death induction in mammary involution, those that would be turned-on by milk stasis upon cessation of suckling, from those dependent on the levels of regulatory factors expressed by the differentiated mammary epithelium, such as TTP. Importantly, our results reveal that inflammatory cytokines in the lactating mammary epithelium are tightly regulated. Particularly, controlling TNFα expression and/or activity appears to be a determinant for defining differentiated mammary cell fate. Finally, our results suggest that, similar to forced weaning, TTP loss triggers signaling pathways that may confer an increased risk for developing breast cancer [39]. Therefore, TTP physiological role during lactation would comprise its most recognized capabilities as a potent anti-inflammatory and tumor-suppressor protein.

## MATERIALS AND METHODS

### Generation of MG-TTP KO mice and *in vivo* experiments

*Ttp*LoxP/LoxP [49] mice were crossed with mice expressing Cre recombinase under the control of the mammary-specific *Wap* promoter [50], both on C57BL/6J backgrounds. Expression of *Wap–Cre* was only detected in alveolar epithelial cells of mammary tissue during lactation [50]. Supplementary Figure 4A shows the program of mating to generate the experimental MG-TTP KO (*Wap-Cre*^*+/-*^*/Ttp*^*LoxP/LoxP*^) and their littermate (*Wap-Cre*^*-/-*^*/Ttp*^*LoxP/LoxP*^) as Control mice. Cre activity results in deletion of exon 2, which encodes the tandem zinc finger domain that is responsible for the RNA binding activity (Figure 1A). Primers used for genotyping are described in Supplementary Table 1. For the experiments, three month-old virgin adult Control (*Wap-Cre*^*-/-*^*/Ttp*^*LoxP/LoxP*^) females were used for establishing the base line of β-casein and TTP expression levels (Figure 1), or mated, as the experimental MG-TTP KO (*Wap-Cre*^*+/-*^*/Ttp* ^*LoxP/LoxP*^) mice. Between days 3-5 postpartum, the number of pups for each lactating female was normalized to six, and at least three lactating females were analyzed per group. Mice were sacrificed in a CO_2_ chamber on days 10 and 15 of lactation, and the 4th mammary gland pair was used for mRNA and protein analysis.

All animal studies were approved by the CICUAL-FCEN-UBA (www.cicual.de.fcen.uba.ar) and conducted in accordance with the NIH Guide for the Care and the Use of Laboratory Animals in a pathogen-free, temperature-controlled environment, on a 12 h/12 h light/dark cycle. Mice were given sterilized laboratory chow and water *ad libitum*.

Treatments with intraperitoneal injection of Etanercept (Enbrel^®^, Wyeth), 0.4 mg per 40 g body weight or vehicle (PBS) were performed 3 times a week for 2 weeks after parturition or 0, 24, 48 h after weaning. Administration regimens were adapted from previous mouse studies [51].

### Morphological and immunohistochemical analysis

Tissues sections were stained with Haematoxylin and Eosin (H&E) or Masson’s trichrome for morphological analysis. For immunohistochemistry, the antibodies used are described in Supplementary Table 2. For quantification, at least 1000 cells were counted in each sample.

### Real-time polymerase chain reactions (RT-qPCR)

RNA was isolated using Trizol (Invitrogen); 5 µg of RNA were reverse transcribed using M-MLV Reverse Transcriptase (Invitrogen). RT-qPCR was performed as previously reported [9]. Primers used are described in Supplementary Table 1. Values were calculated by the Standard curve method [52].

### Immunoblotting

Total protein extracts for Western blotting (WB) were prepared as described previously [52]. Blots were probed with the antibodies described in Supplementary Table 2. Densitometry analysis was performed using ImageJ 1.34s software (Wayne Rasband, NIH, http://rsb.info.nih.gov/ij/).

### Statistical analysis

Two-way ANOVA followed by Tukey’s Multiple Comparisons Test or Student’s *T* test (to make comparisons between two groups) were performed using GraphPad Prism-5 (GraphPad Software). Differences were considered statistically significant if *p* ≤ 0.05.

## SUPPLEMENTARY MATERIALS FIGURES AND TABLES


