# Supplementary material for: Expression of the mRNA stability regulator Tristetraprolin is required for lactation maintenance in the mouse mammary gland
Source: Oncotarget. 2018 Jan 3;9(9):8278–89. doi: 10.18632/oncotarget.23904 (PMC5823555; doi:10.18632/oncotarget.23904)
Supplement: Supplementary file 1 [file oncotarget-09-8278-s001.pdf]

Expression of the mRNA stability regulator Tristetraprolin is required for lactation maintenance in the mouse mammary gland

SUPPLEMENTARY MATERIALS

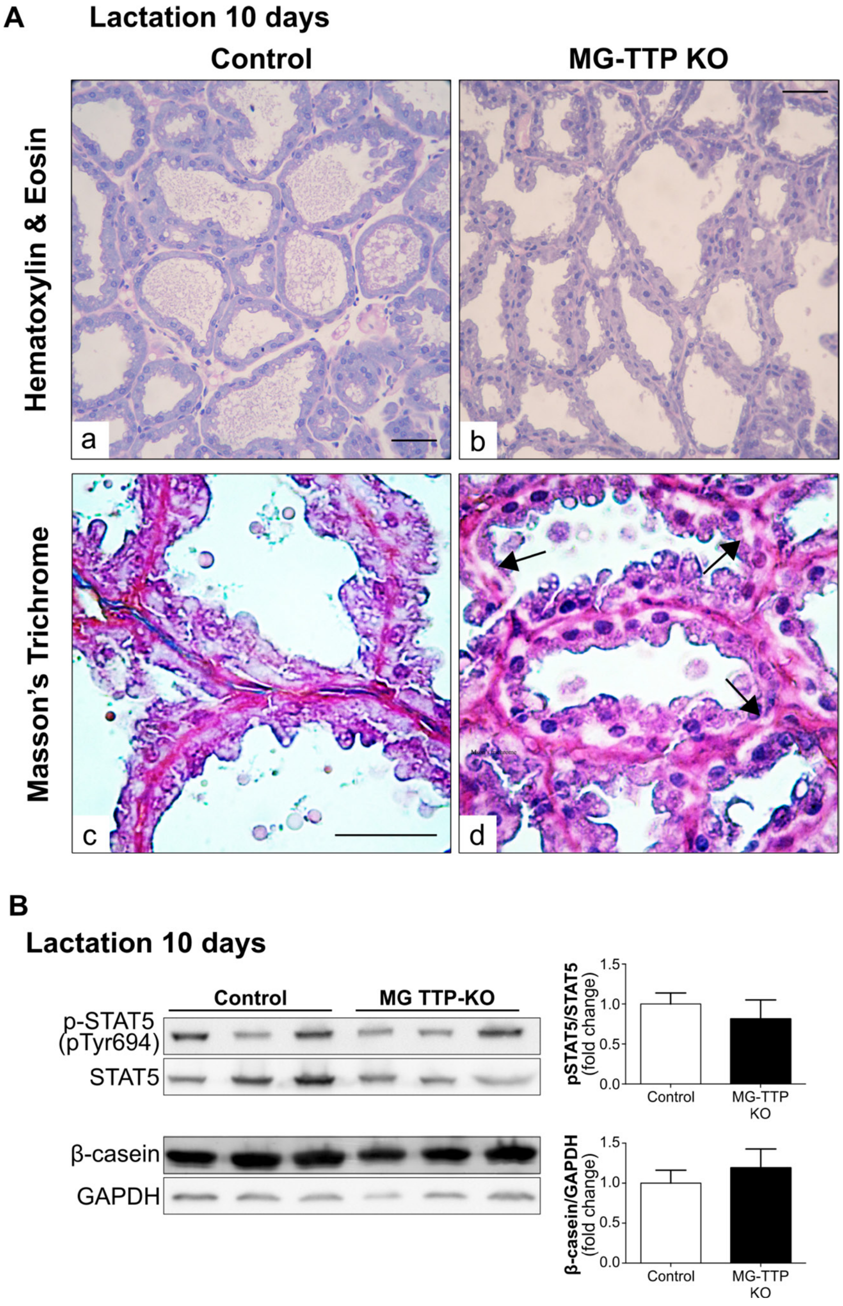

Supplementary Figure 1: MG-TTP KO and control mammary glands at 10 days of lactation. (**A**) Histological analysis: a&b show representative images of H&E stained mammary gland sections, original magnification: 400X; c&d show representative images of Masson's tri-chromic stained sections; arrows point out regions of apparent basement membrane thinness, original magnification 1000X. Scale bar 100 µm. (**B**) Representative images and quantification of WB analysis from Control and MG-TTP KO mammary glands with the indicated antibodies. Each column shows mean + s.e.m. ($n = 3$).

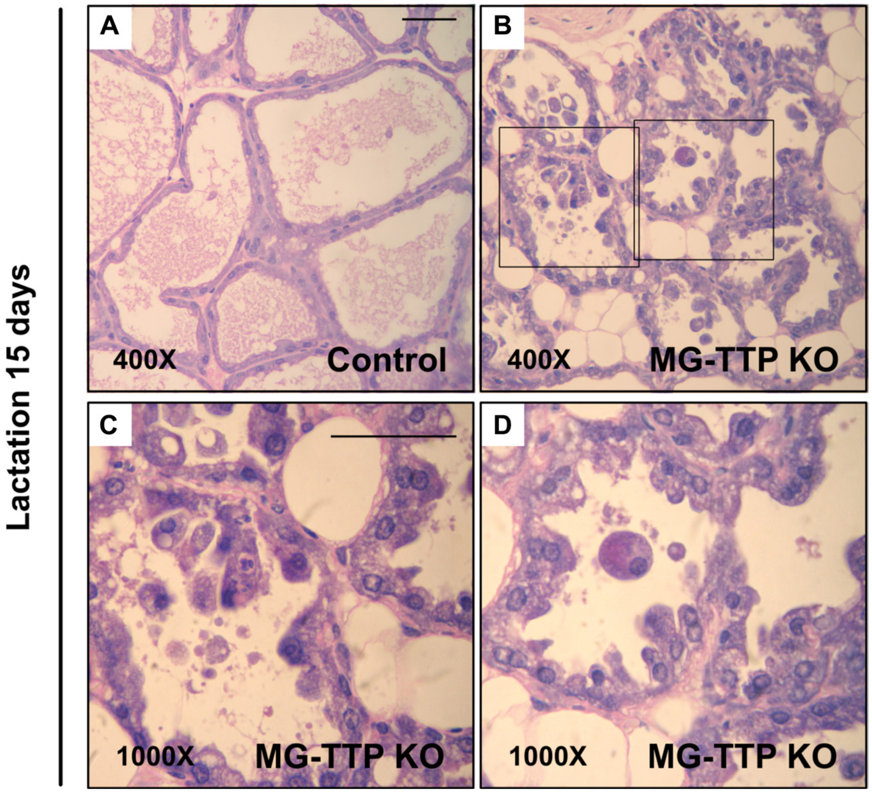

Supplementary Figure 2: Eliminate Representative images of H&E stained mammary gland sections from Control (**A**) and MG-TTP KO (**B, C** and **D**) mice at 15 days of lactation. Original magnifications are shown in the lower left bottom of each image. C&D show framed areas in B. Scale bar 100 μm.

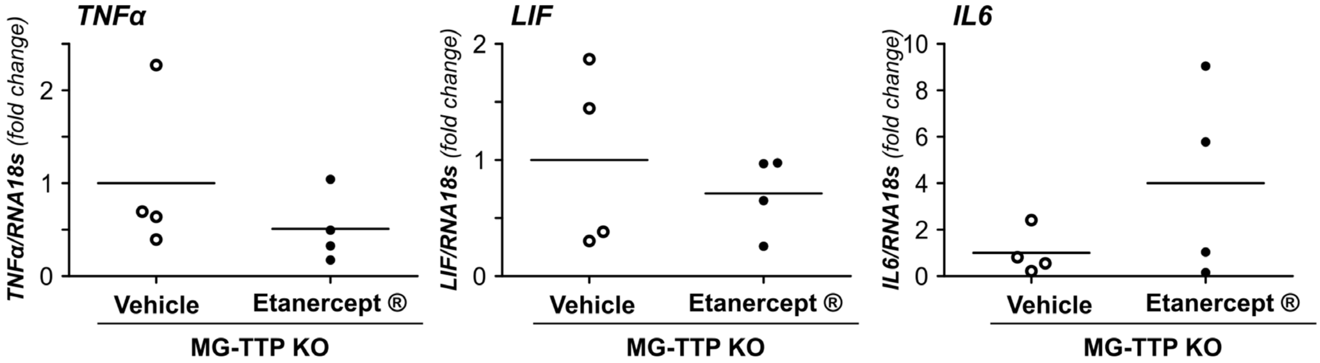

Supplementary Figure 3: Inflammatory cytokine mRNA levels in 15 day lactating MG-TTP KO mice treated with vehicle or etanercept. RT-qPCR analysis of TNFα, LIF and IL6 levels normalized to 18S RNA; fold changes compared to vehicle are shown. Each graph of statistical dot plots shows the median percent (black bars). Each point corresponds to a different mouse.

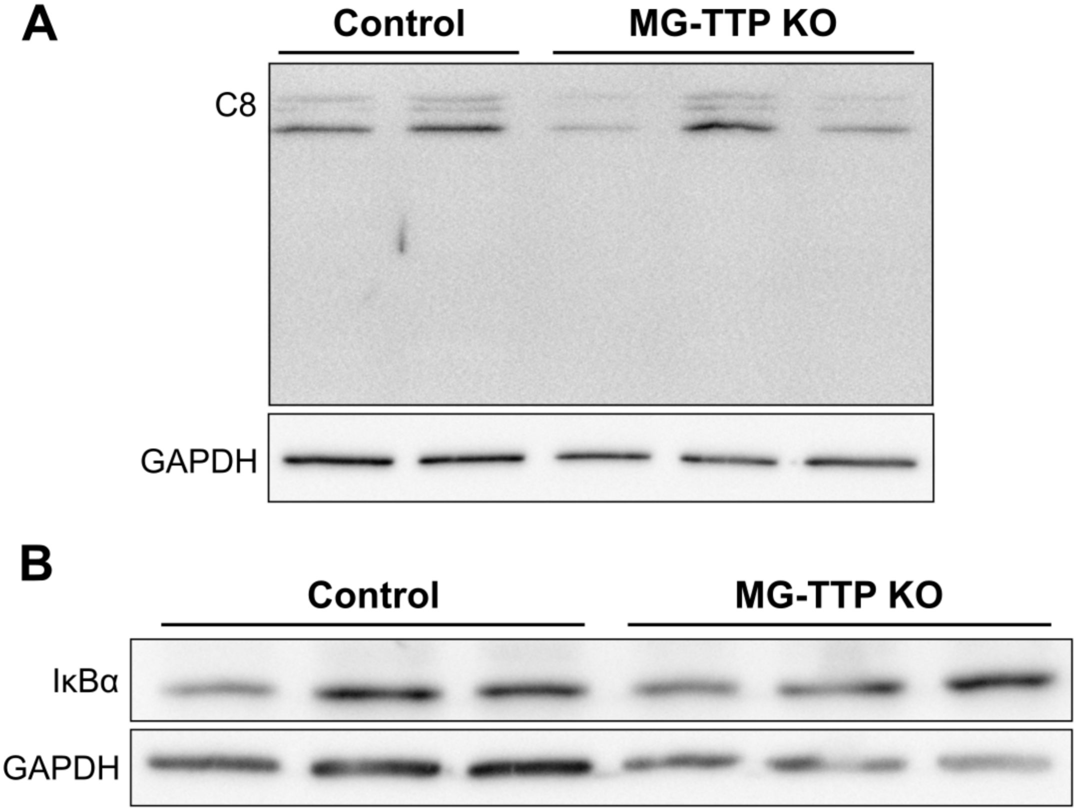

Supplementary Figure 4: Representative images of Western Blot analysis from Control and MG-TTP KO mammary glands at 15 days of lactation with antibodies against caspase 8 (C8) (**A**) and IkB alpha (**B**).

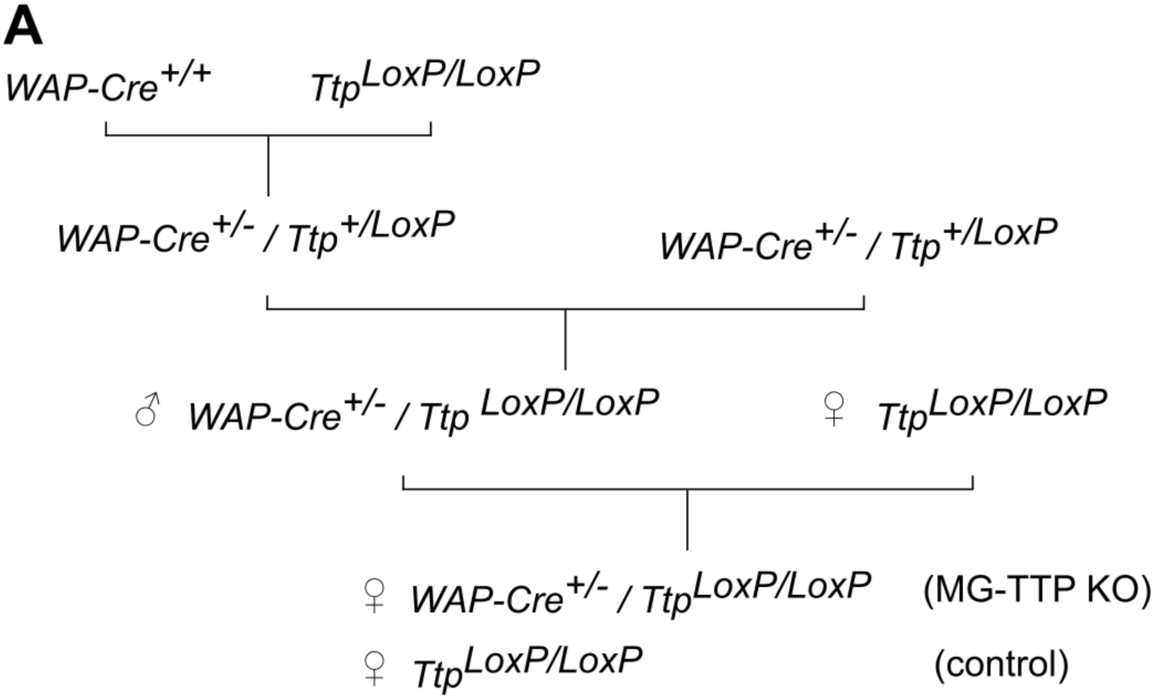

Supplementary Figure 5: Generation of mammary gland-specific TTP deficient mice. (**A**) Breeding scheme to generate MG-TTP KO and *Cre*$^{-}$ control mice. *Ttp*$^{LoxP/LoxP}$ were mated with *Wap-Cre*$^{+/+}$ to generate *Wap-Cre*$^{+/-}$/*Ttp*$^{+}$/^{LoxP} bitransgenic mice. Intercrosses of *Wap-Cre*$^{+/-}$/*Ttp+*/^{LoxP} resulted in *Wap-Cre*$^{+/-}$/Ttp $^{LoxP/LoxP}$ mice. Then, *Wap-Cre*$^{+/-}$/Ttp $^{LoxP/LoxP}$ males were backcrossed with *TTP loxP/loxP* females to generate *Wap-Cre*$^{+/-/}$Ttp$^{LoxP/LoxP}$ (MG-TTP KO) and *Ttp*$^{LoxP/LoxP}$ (Control).

Supplementary Table 1: PCR primers

Genotyping	Primers	Amplicon length
Ttp LoxP/LoxP (P1 and P2) [1]	P1 - Fw 5'- GAA CCC TCT CTC GAT CGG GGA TAC -3'<br>P2 - Rv 5' -GGA TGG AGT CCG AGT TTA TGT TCC AA -3'	WT<br>327 bp<br>Ttp LoxP/LoxP<br>514 bp
Wap-Cre+/- [2]	Fw 5'- TAG AGC TGT GCC AGC CTC TTC -3'<br>Rv 5'- CAT CAC TCG TTG CAT CGA CC -3'	Cre : 210 bp
Wap-Cre+/-/Ttp LoxP/ LoxP (P3 - P2 - P4) [1]	P3 - Fw 5'- CTG GCT GGA AAT GAG AGA GG -3'<br>P2 - Rv 5'- GGA TGG AGT CCG AGT TTA TGT TCC AA -3'<br>P4 - Rv 5'- CAC CCC TTA CGC CAG AAC TA -3'	Cre-Deleted TtpLoxP/ LoxP:<br>769 bp<br>Ttp LoxP/LoxP<br>870 bp
RT-qPCR		
18s RNA	Fw 5'- GTA ACC CGT TGA ACC CCA TT -3'<br>Rv 5'- CCA TCC AAT CGG TAG TAG CG -3'	151 bp
β-csn [3]	Fw 5'- GAT GCC CCT CCT TAA CTC TGA A -3'<br>Rv 5'- TTA GCA AGA CTG GCA AGG CTG -3'	75 bp
GAPDH	Fw 5'- AGA AGG TGG TGA AGC AGG CAT C -3'<br>Rv 5'- CGA AGG TGG AAG AGT GGG AGT TG -3'	111 bp
IL-6 [4]	Fw 5'- GAG GAT ACC ACT CCC AAC AGA CC -3'<br>Rv 5'- AAG TGC ATC ATC GTT GTT CAT ACA -3	141 bp
Lif [5]	Fw 5'- GGC AAC CTC ATG AAC CAG ATC A -3'<br>Rv 5'- GCA AAG CAC ATT GCT GAG GAG G -3'	336 bp
TNFα [5]	Fw 5'-AGC CGA TTT GCT ATC TCA TAC C-3´<br>Rv 5'-AGT ACT TGG GCA GAT TGA CCT C-3´	184 bp
Ttp	Fw 5'- CGG AAC TCT GCC ACA AG -3'<br>Rv 5'- GGC GAA AAG GAA CAA GA -3'	214 bp
Wap	Fw 5'- CCA GCG ACC GTG AGT GTT C -3'<br>Rv 5'- GGA GTG AAG GGT CTT GCT GTA TAG -3'	166 bp

Supplementary Table 2: Antibodies for Western Blot and Immunohistochemistry (IHC)

Antibody	Company	Technique/Dilution
AKT	Santa Cruz Biotech, sc-1618	Western Blot<br>1:1000
P-AKT (pSer473)	Cell Signaling, Cat#4060	Western Blot<br>1:1000
B-casein	Rabbit Polyclonal antibody generated against whole mouse milk [6].	Western Blot<br>1:10,000
Cathepsin-L	R&D Systems<br>Cat MAB9521	Western Blot<br>1:1000
Cleaved-Caspase 3	Cell Signaling, Cat#9661	IHC<br>1:100
Caspase 8	Santa Cruz Biotech, sc-7890	Western Blot<br>1:500
GAPDH	Santa Cruz Biotech, sc-20357	Western Blot<br>1:5000
IκB	Cell Signaling, Cat#9242S	Western Blot<br>1:1000
STAT3	Cell Signaling, Cat#9145	Western Blot<br>1:1000
P-STAT3 (pTyr705)	Cell Signaling, Cat9145	Western Blot<br>1:800<br>IHC<br>1:100
STAT5	Abcam, Cat.ab32043	Western Blot<br>1:1000
P-STAT5 (pY694)	Cell Signaling, Cat#9351	Western Blot<br>1:800
TTP	SIGMA-Aldrich, T5327	Western Blot<br>1:500

REFERENCES

1. Qiu LQ, Stumpo DJ, Blackshear PJ. Myeloid-specific tristetraprolin deficiency in mice results in extreme lipopolysaccharide sensitivity in an otherwise minimal phenotype. J Immunol. 2012; 188: 5150-9. http://doi.org/10.4049/jimmunol.1103700.
2. Wagner KU, Wall RJ, St-Onge L, Gruss P, Wynshaw-Boris A, Garrett L, Li M, Furth PA, Hennighausen L. Cre-mediated gene deletion in the mammary gland. Nucleic Acids Res. 1997; 25: 4323-30.
3. Nakasato M, Shirakura Y, Ooga M, Iwatsuki M, Ito M, Kageyama S, Sakai S, Nagata M, Aoki F. Involvement of the STAT5 signaling pathway in the regulation of mouse preimplantation development. Biol Reprod. 2006; 75: 508-17. http://doi.org/10.1095/biolreprod.105.047860.
4. Grenon SM, Jeanne M, Aguado-Zuniga J, Conte MS, Hughes-Fulford M. Effects of gravitational mechanical unloading in endothelial cells: association between caveolins, inflammation and adhesion molecules. Sci Rep. 2013; 3: 1494. http://doi.org/10.1038/srep01494.
5. Schere-Levy C, Buggiano V, Quaglino A, Gattelli A, Cirio MC, Piazzon I, Vanzulli S, Kordon EC. Leukemia inhibitory factor induces apoptosis of the mammary epithelial cells and participates in mouse mammary gland involution. Exp Cell Res. 2003; 282: 35-47.
6. Lee EY, Lee WH, Kaetzel CS, Parry G, Bissell MJ. Interaction of mouse mammary epithelial cells with collagen substrata: regulation of casein gene expression and secretion. Proc Natl Acad Sci U S A. 1985; 82: 1419-23.
